# Imaging of Clear Cell Renal Carcinoma with Immune Checkpoint Targeting Aptamer-Based Probe

**DOI:** 10.3390/ph15060697

**Published:** 2022-06-01

**Authors:** Stanisław Malicki, Barbara Pucelik, Edyta Żyła, Małgorzata Benedyk-Machaczka, Wojciech Gałan, Anna Golda, Alicja Sochaj-Gregorczyk, Marta Kamińska, João Crispim Encarnação, Barbara Chruścicka, Hans-Peter Marti, Tony Jialiang Chen, Katarzyna Magiera-Mularz, Bartosz Zięba, Tad A. Holak, Janusz M. Dąbrowski, Anna Czarna, Joanna Kozieł, Piotr Mydel, Grzegorz Dubin

**Affiliations:** 1 Malopolska Centre of Biotechnology, Jagiellonian University, Gronostajowa 7a, 30-387 Krakow, Poland; barbara.pucelik@uj.edu.pl (B.P.); edyta.e.zyla@gmail.com (E.Ż.); alicja.sochaj-gregorczyk@uj.edu.pl (A.S.-G.); bartek.zieba@gmail.com (B.Z.); anna1.czarna@uj.edu.pl (A.C.); 2 Department of Microbiology, Faculty of Biochemistry, Biophysics and Biotechnology, Jagiellonian University, Gronostajowa 7, 30-387 Krakow, Poland; malgorzata.benedyk@uj.edu.pl (M.B.-M.); anna.b.golda@uj.edu.pl (A.G.); barbara.chruscicka-smaga@uj.edu.pl (B.C.); joanna.koziel@uj.edu.pl (J.K.); 3 Department of Computational Biophysics and Bioinformatics, Faculty of Biochemistry, Biophysics and Biotechnology, Jagiellonian University, Gronostajowa 7, 30-387 Krakow, Poland; wojciech.galan@gmail.com; 4 Broegelmann Research Laboratory, Department of Clinical Science, University of Bergen, N-5021 Bergen, Norway; marta_kaminska@outlook.com; 5 Ridgeview Instruments AB, 75237 Uppsala, Sweden; joaocrispim90@gmail.com; 6 Department of Clinical Medicine, University of Bergen, N-5021 Bergen, Norway; hans-peter.marti@uib.no (H.-P.M.); tony.chen@uib.no (T.J.C.); 7 Faculty of Chemistry, Jagiellonian University, Gronostajowa 2, 30-387 Krakow, Poland; k.magiera@uj.edu.pl (K.M.-M.); holak@chemia.uj.edu.pl (T.A.H.); jdabrows@chemia.uj.edu.pl (J.M.D.)

**Keywords:** immune checkpoint, PD-L1, aptamer, imaging, cancer

## Abstract

Immune checkpoint targeting immunotherapy has revolutionized the treatment of certain cancers in the recent years. Determination of the status of immune checkpoint expression in particular cancers may assist decision making. Here, we describe the development of a single-stranded aptamer-based molecular probe specifically recognizing human PD-L1. Target engaging aptamers are selected by iterative enrichment from a random ssDNA pool and the binding is characterized biochemically. Specificity and dose dependence is demonstrated in vitro in the cell culture using human kidney tumor cells (786-0), human melanoma cells (WM115 and WM266.4) and human glioblastoma LN18 cancer cells. The utility of the probe in vivo is demonstrated using two mouse tumor models, where we show that the probe exhibits excellent potential in imaging. We postulate that further development of the probe may allow universal imaging of different types of tumors depending on their PD-L1 status, which may find utility in cancer diagnosis.

## 1. Introduction

Biomedical imaging, one of the pillars of modern cancer management, is tightly integrated into clinical decision making. Imaging is involved in all stages of the diagnostic process including screening, staging, therapy planning and management. Early diagnosis is known to be a major factor in the reduction of mortality, treatment costs and hospital stays. Since cancer is a multi-factorial disease multiple imaging approaches need to be combined to correspond to various mechanisms and phases of development. With the use of highly specific probes, it is now possible to visualize cancer development at a very early stage [1,2].

In the last two decades, monoclonal antibodies (mAbs) have become an increasingly successful treatment modality in a wide range of diseases due to their high specificity and affinity. The same properties of mAbs are also explored in molecular imaging to target antigens of interest in vivo [3]. Imaging probes are the key elements of molecular imaging and have to offer high sensitivity, low background noise, low toxicity and relative stability [4]. A number of studies indicate that, in certain approaches, aptamers are superior to mAbs offering reduced immunogenicity, structural stability and smaller size (~ 3 nm compared to 10–15 nm for antibodies) [5]. However, to date the use of aptamers in imaging has not been sufficiently explored.

Aptamer-based probes were shown to provide superior images as compared to mAbs in stimulated emission depletion microscopy (STED) [6] and were able to recognize more epitopes than antibodies; thus, providing a denser labeling of the structures of interest. In addition, a study comparing the utility of ^111^In-labeled aptamers and antibodies targeting EGFR in μSPECT/CT imaging demonstrated better tumor-specific uptake of aptamers in nude mice bearing the highly malignant human OSC-19 tumors [7]. These two studies clearly demonstrated the superior quality of aptamer-based probes over mAbs in terms of obtaining accurate images for cell biology and in vivo. Consequently, aptamers are evaluated as diagnostic tools in a number of clinical trials [8]. Nonetheless, the diversity of available diagnostic imaging techniques employed in tumor detection, characterization and evaluation of therapeutic interventions (optical imaging, fluorescence and bioluminescence, magnetic resonance imaging (MRI), positron-emission tomography (PET), single-photon emission computed tomography (SPECT), computed tomography (CT), and ultrasound (US) techniques and vast landscape of possible markers opens a significantly broader and yet unexplored perspective of the utility of aptamer-based probes [8].

Here, we describe the development of a single-stranded aptamer-based probe specific for human Programmed death-ligand 1 (PD-L1). SELEX methodology allowed to identify functional oligonucleotide sequence, which was then examined for specificity and ability to selectively label tumor cells in several in vitro and in vivo models. Most interestingly, our probe demonstrated the potential of in vivo tumor imaging in two unrelated mouse models providing a starting point for the development of the noninvasive diagnostic method of tumor classification according to the PD-L1 status.

## 2. Results

### 2.1. Selection of Aptamers

**Figure 1:**
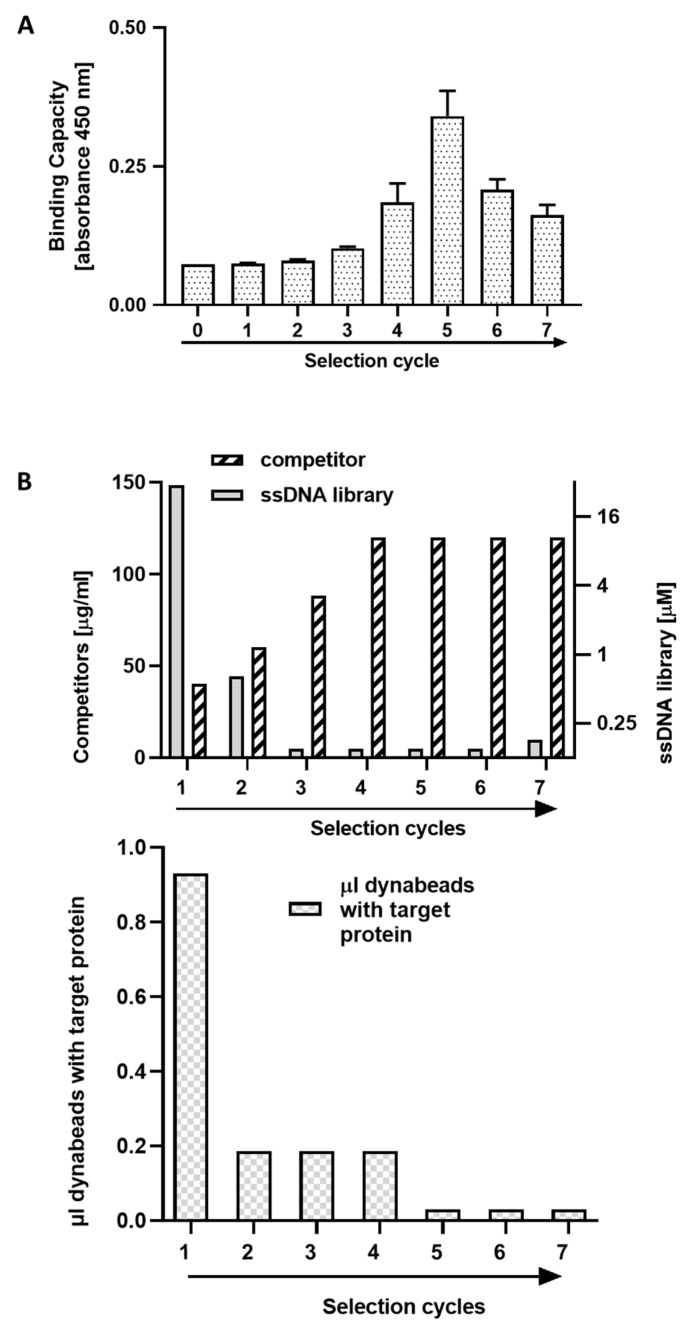
Selection of PD-L1 targeting aptamers. (**A**) Enrichment of ssDNA pool with aptamers recognizing the extracellular domain of PD-L1 after indicated selection cycles, determined using ELISA (binding of biotinylated ssDNA pool to immobilized target protein). (**B**) Gradual increase in selection pressure at subsequent selection cycles was obtained by increasing the concentration of competitor (ytRNA) while simultaneously decreasing the concentration of ssDNA library and target protein.

Aptamers recognizing the IgV extracellular domain of PD-L1 (residues 18–134) were selected using the SELEX procedure where the ssDNA library was enriched towards the target protein immobilized on magnetic beds (Figure 1). Gradual increase in selection pressure applied within the standard SELEX procedure [9], allowed to significantly limit the number of selection cycles leading to convergence, consistently with the previously described methodology [9,10]. Saturation of ssDNA pool binding to the target protein was observed already in the 5th selection cycle as determined by ELISA (Figure 1A). Subsequent selection cycles resulted in a decrease in the level of binding of the ssDNA pool. This unexpected effect was explained in post analysis by excessive amplification of sequences with low affinity to PD-L1 presumably related to positive selection within the amplification (and not the binding) step (see below).

### 2.2. Bioinformatic Analysis (Clustering) of Aptamers

**Figure 2:**
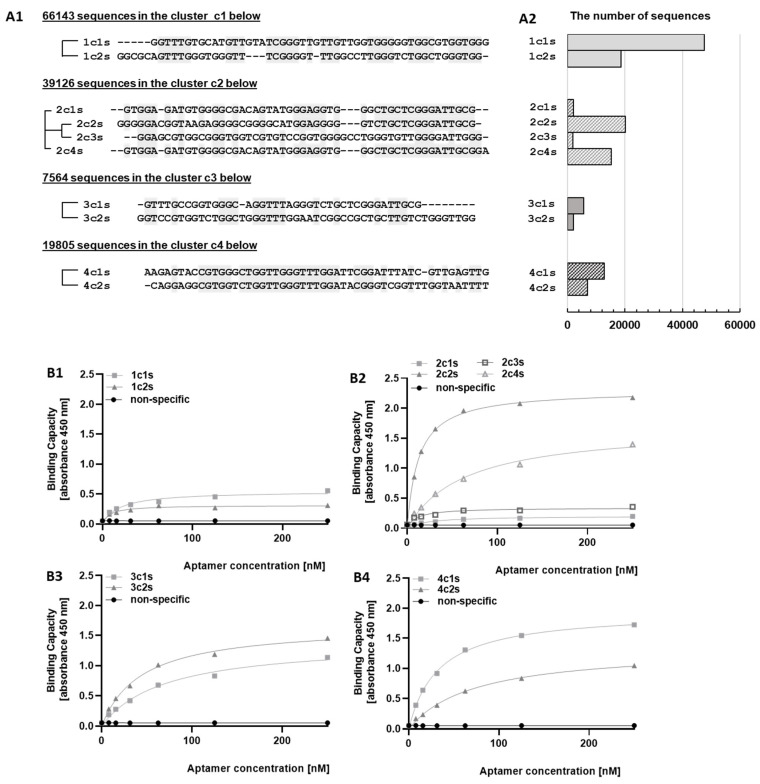
Aptamer analysis after the last (7th) selection cycle. (**A1**) (pseudo-) phylogenetic trees generated based on the multiple sequence alignment (MSA) of sequences belonging to indicated clusters. Trees were created for clusters of >2 sequences. (**A2**) number of sequences in groups shown in A1 (**B1**–**B4**) ELISA was used to determine the binding capacity of representative aptamers selected within each cluster towards PD-L1.

The pool of aptamers obtained after the 5th, 6th and 7th selection cycle was analyzed by sequencing (Figure 2). Bioinformatic analysis identified 10 frequently repeating sequences which have been grouped into four clusters by sequence similarity (Figure 2A1). The amplified sequences did not differ qualitatively between analyzed selection cycles (all dominating sequences present after the 5th cycle were also present after the 7th cycle; data not shown). Quantitatively, however, the abundance of 1c cluster increased gradually along with the selection cycles (from low abundance in cycle 5 to high abundance in cluster 7; Supplementary Figure S3).

### 2.3. Binding of Selected Aptamers to PD-L1

The most abundantly represented sequences were obtained in biotinylated form and the binding to PD-L1 was analyzed by ELISA. Aptamers belonging to clusters 3c and 4c and some aptamers belonging to cluster 2c demonstrated strong interaction with the extracellular domain of PD-L1 (Figure 2B2–B4), while the interaction of aptamers belonging to cluster 1c was weak (Figure 2B1). The fact that the component of cluster 1c increased gradually from cycle 5 to cycle 7 (Figure S2) explains the overall decrease in the affinity of the ssDNA pool in the referenced cycles (Figure 1A). We reason that such an effect may be caused by preferential amplification of sequences belonging to cluster 1 in PCR reaction, for example, due to the high thermal stability of the secondary structures within other clusters. However, these effects were not studied systematically.

**Figure 3:**
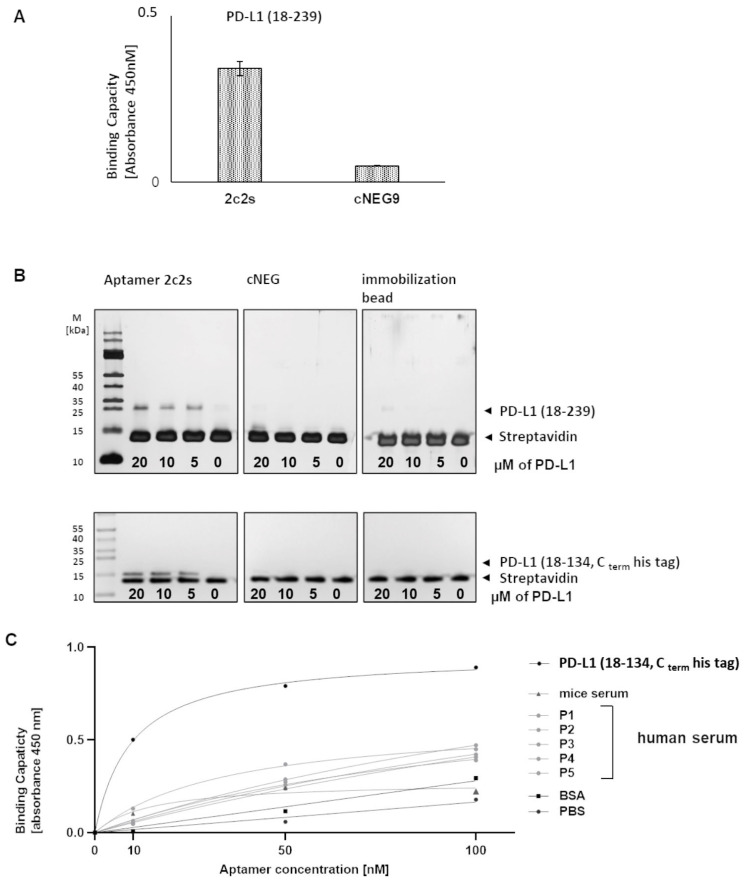
The binding capacity of 2c2s to PD-L1. (**A**) ELISA mediated detection of aptamer bound to immobilized PD-L1; (**B**) protein immobilization assay. Indicated, biotinylated aptamers were immobilized on streptavidin-coated beads and retention of PD-L1 was assessed by SDS-PAGE. (**C**) ELISA mediated detection of 2c2s interaction with indicated protein coated surface.

Aptamer 2c2s was selected for further analysis because it was characterized by the strongest ELISA signal and occurred at the highest frequency among strongly interacting sequences after each of the last three selection cycles (Figure 2A2, Supplementary Figure S1). First, the affinity of 2c2s to an alternative construct of PD-L1 (amino acids 18–239) was evaluated in ELISA format (Figure 3A). Additionally, the affinity of 2c2s to PD-L1 was verified in an alternative assay format: the PD-L1 protein immobilization test (Figure 3B). In both assays, 2c2s was characterized by a significantly higher signal level compared to the nonspecific ssDNA sequence, used as negative control, indicating that the observed affinity of 2c2s for PD-L1 is assay and protein construct independent (Figure 3B).

Selectivity of 2c2s was evaluated by comparing its binding capacity to PD-L1 with that to nontarget proteins including bovine albumin, mouse and human serum in ELISA format. Wells coated with PD-L1 accumulated significantly higher levels of 2c2s compared to the wells coated with nontarget proteins demonstrating selective binding of 2c2s to PD-L1 (Figure 3C).

### 2.4. Detection of PD-L1 at the Cell Surface

**Figure 4:**
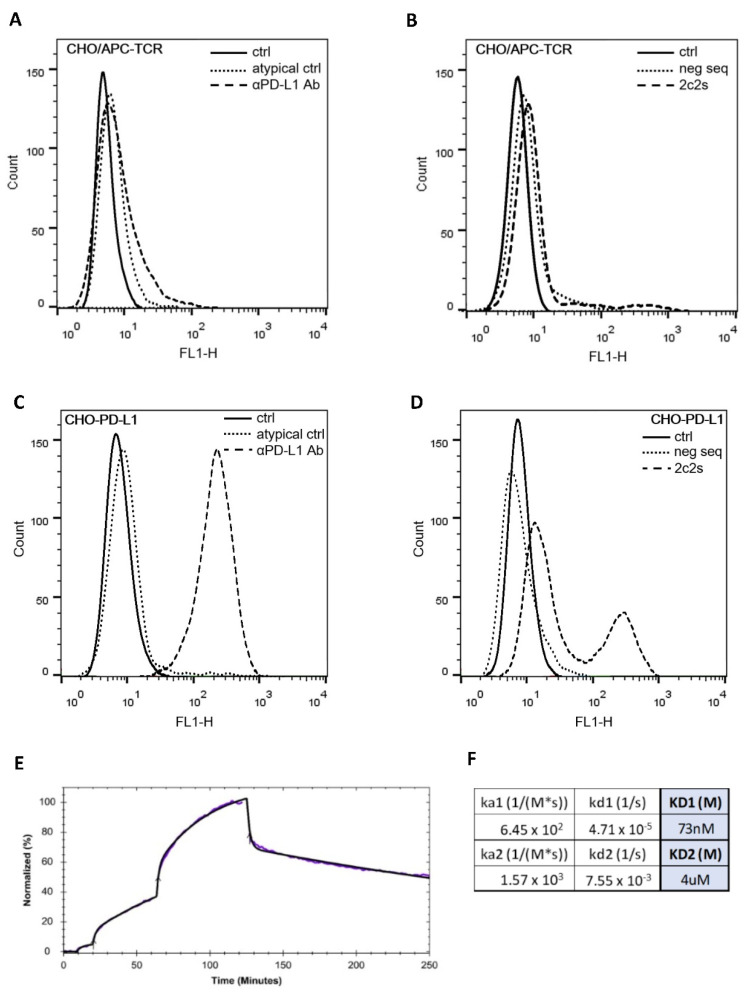
The 2c2s specifically labels PD-L1 at the cell surface. CHO/APC-TCR cells were labeled with (**A**) α-PD-L1 mAb (clone MIH1) or (**B**) FAM conjugated 2c2s, and CHO-PDL1 cells (overexpressing PD-L1) were labeled with (**C**) α-PD-L1 mAb (clone MIH1) or (**D**) FAM conjugated 2c2s, and evaluated by flow cytometry. Isotype antibody and scrambled aptamer served as controls, respectively. (**E**) Ligand Tracer binding traces. Model fit to experimental data suggests two interaction sites with parameters given in panel (**F**).

To initially assess the utility of 2c2s to detect PD-L1 in the context of the living cell, aAPC/CHO-K1 cells or PD-L1 aAPC/CHO-K1 cell lines stably overexpressing the target protein were used. The cells were stained with fluorescently labeled 2c2s and analyzed by flow cytometry. First, the target cells were confirmed to uniformly express PD-L1 as demonstrated by homogenous labeling with α-PD-L1 antibody (Figure 4C). Fluorescently labeled scrambled aptamer did not show binding to the cells expressing PD-L1 (Figure 4D). Fluorescently labeled 2c2s aptamer distinguished two populations of cells: a strongly and weakly labeled ones (Figure 4D). The effect was consistent over multiple experiments (not shown). aAPC/CHO-K1 cells were not significantly labeled by the antibody or 2c2s (Figure 4A). Such results indicate that 2c2s recognizes a different epitope at PD-L1 compared to the antibody and that the 2c2s recognized epitope is partially masked at a subpopulation of cells. Unlike the antibody, 2c2s was unable to dissociate the PD-1/PD-L1 at the cell surface (Figure S3) as tested using reporter assay described previously [11,12,13], again demonstrating that the antibody and 2c2s recognize different epitopes at PD-L1.

Further, we evaluated the binding kinetics of 2c2s to PD-L1 at the cell surface using Ligand Tracer (Figure 4E). Time traces of ligand accumulation at immobilized cells were acquired. The best fitting of experimental data was obtained using a two-site interaction model with a strong affinity site characterized by K_d_ of 73 nM and a second, significantly weaker site characterized by K_d_ of 4 µM, which latter site we interpreted as unspecific binding (Figure 4F).

**Figure 5:**
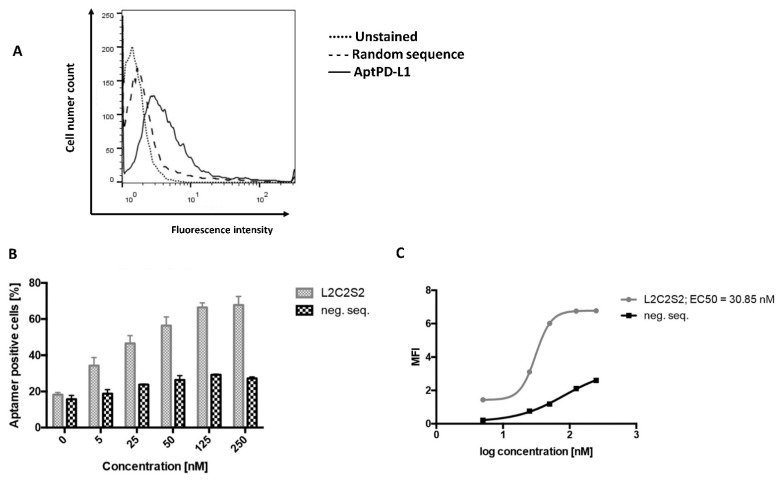
Interaction of 2c2s with human PD-L1 at the cell surface. Renal adenocarcinoma cells (786-0) were incubated for 30 min at 37 °C with indicated concentrations of FITC-labeled 2c2s and the interaction was analyzed by flow cytometry. (**A**) Representative histogram and (**B**) mean percentage of 2c2s-labeled 786-0 cells. Data are expressed as the mean ± SD of three independent experiments. (**C**) The affinity of 2c2s to PD-L1 at the cell surface determined using data in panel B.

To further evaluate 2c2s potential in detecting PD-L1 at the cell surface, renal adenocarcinoma cell line, 786-0, characterized by physiological expression of PD-L1 [14] was used. The cells were treated with FITC-labeled 2c2s or FITC-conjugated nonspecific ssDNA control and analyzed by flow cytometry. 2c2s specifically labeled 786-0 cells while control aptamer with a nonspecific sequence was significantly less active (specificity window of 0.5 log concentration; Figure 5A). The level of 2c2s labeling was dose-dependent with EC_50_~31nM (Figure 5B,C), comparable to K_d_ of 73nM determined with PD-L1 aAPC/CHO-K1cells. Unlike PD-L1 aAPC/CHO-K1 cells, the 786-0 cells were uniformly labeled suggesting that epitope masking was PD-L1 aAPC/CHO-K1 specific.

**Figure 6:**
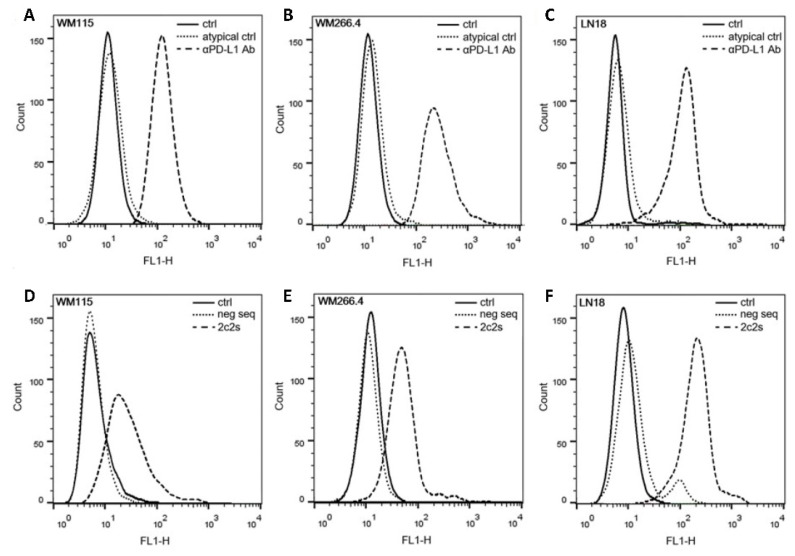
Detection of PD-L1 expression at the surface of representative melanoma cell lines by flow cytometry. (**A**,**D**) WM115, (**B**,**E**) WM266.4 and (**C**,**F**) LN18. Top panels—mAb staining (50 nM); bottom panels—2c2s staining (50 nM). Isotype antibody and scrambled sequence aptamer were used as respective controls.

Partial masking of 2c2s epitope on PD-L1 overexpressed in PD-L1 aAPC/CHO-K1 cells prompted us to further characterize the recognition of PD-L1 physiologically expressed on tumor cells by 2c2s. Evaluation of a panel of melanoma cell lines by staining with α-PD-L1 mAb and 2c2s demonstrated that only some melanomas are characterized by significant PD-L1 expression which agrees with prior studies. Importantly, α-PD-L1 mAb staining (Figure 6A,B) correlated with 2c2s labeling (Figure 6D,E) and positive cells labeled uniformly both with 2c2s and the antibody with no indication of epitope masking.

Similar results were obtained when a panel of glioma cell lines were evaluated. Again, only selected cell lines expressed a detectable amount of PD-L1, the strong correlation between 2c2s and α-PD-L1 mAb labeling was observed and the labeling of positive cells was uniform in each tested case (Figure 6 C,F). Curiously, we observed that a residual population of LN18 cells stained with scrambled sequence aptamer (Figure 6F), but the fact was not systematically investigated beyond the observation only.

Overall, the above results indicate that 2c2s specifically recognizes PD-L1 expressed at the cell surface. Partial epitope masking was observed only upon ectopic overexpression of human antigen in the murine cell line and is not relevant to physiological expression in cancer cell lines tested.

### 2.5. 2c2s as a Probe for Cancer Imaging Ex Vivo

**Figure 7:**
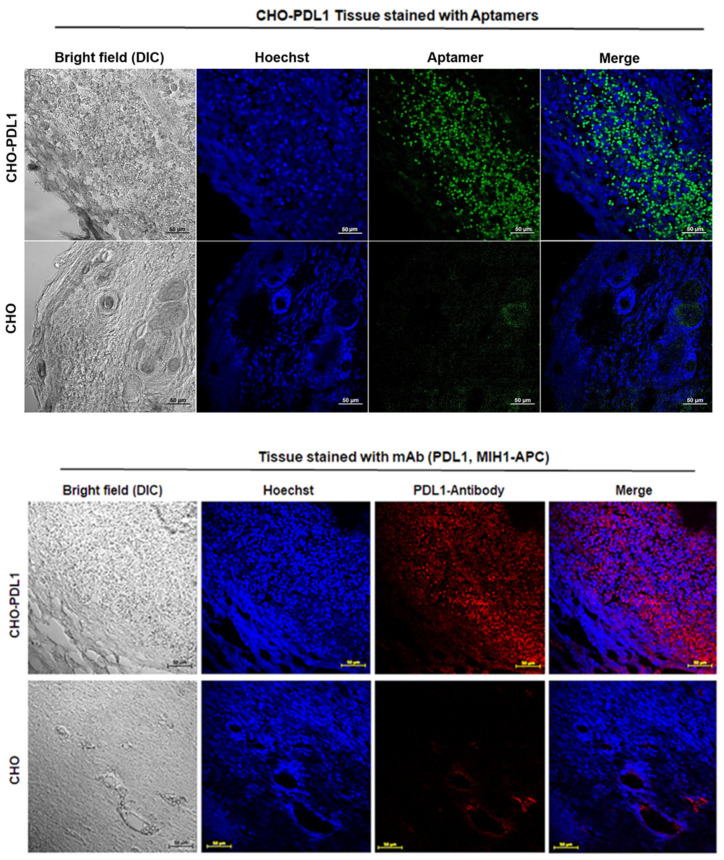
Ex vivo detection of PD-L1 expression in tumor tissue. Tumors formed in mice inoculated with aAPC/CHO-K1 and PD-L1 aAPC/CHO-K1 cells were excised, labeled with 2c2s-FAM (**top** panel) or control PD-L1 antibody (MIH1, **bottom** panel) and imaged by confocal fluorescence microscopy.

To determine the specificity and utility of 2c2s in cancer imaging ex vivo, animals were subcutaneously inoculated with aAPC/CHO-K1 cells or PD-L1 aAPC/CHO-K1 cells (Figure S5). The tumors were allowed to reach a detectable size, excised, labeled with 2c2s or control antibody (MIH1), and imaged by fluorescent microscopy. Upon 2c2s labeling strong signal was observed from PD-L1 overexpressing cells while tumors formed of aAPC/CHO-K1 cells were not labeled (Figure 7, top panel), demonstrating that 2c2s allows selective detection of PD-L1 in histological tumor staining. A comparable staining pattern was obtained with α-PD-L1 mAbs, further supporting the specificity of 2c2s labeling (Figure 7, bottom panel).

### 2.6. 2c2s Aptamer as a Probe for Enhanced In Vivo Cancer Imaging

**Figure 8:**
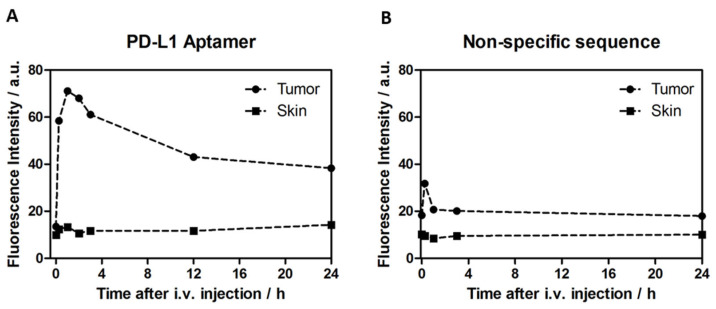
In vivo noninvasive tumor imaging with 2c2s-Cy5.5. PD-L1 aAPC/CHO-K1 (PD-L1 overexpressing) tumor bearing mice were injected intravenously with (**A**) Cy5.5-labeled 2c2s or (**B**) Cy5.5-labeled unspecific aptamer, and the fluorescence signal was monitored through the skin at tumor and adjacent sites.

Animals bearing PD-L1 aAPC/CHO-K1 subcutaneous tumors were intravenously injected with Cy5.5-labeled 2c2s and fluorescence was monitored through the skin using a point monitoring device (Figure S6). The specific fluorescence from the tumor and from skin distant to the palpable tumor were collected at consecutive time points (Figure 8). A strong signal was obtained from the PD-L1 aAPC/CHO-K1 tumor site compared to an almost undetectable signal from skin distant from the palpable tumor site (Figure 8A). Signal was slightly increased at the tumor site compared to the background when labeled scrambled DNA was used instead of a specific probe, demonstrating that tumors unspecifically accumulate labeled short DNA, likely through filtration via leaky vessels (Figure 8B). Nonetheless, the signal from the PD-L1-targeting aptamer was significantly stronger until 24 h post injection compared to that from scrambled control. Additionally, the signal from the PD-L1-targeting aptamer was stronger at the tumor site compared to adjacent skin through the entire experiment. The above data demonstrate the utility of 2c2s for specific, noninvasive tumor identification in vivo.

**Figure 9:**
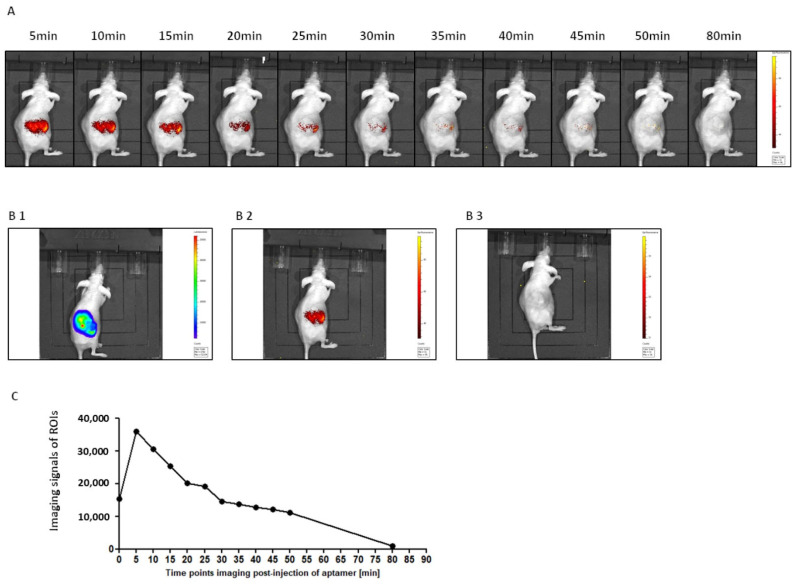
Tumor imaging using 2c2s probe. PD-L1-specific ssDNA aptamer probe (Cy5.5-2c2s) was administered intraperitoneally into Luc-ccRCC tumor bearing animals. (**A**) Fluorescence imaging scans were recorded at indicated time points. (**B**) Co-localization of bioluminescence (**B1**) and fluorescence (**B2**) 10 min after the aptamer injection. Labeled unspecific aptamer does not accumulate in the tumor (**B3**). (**C**) Timeline of fluorescence intensity at the tumor site—numerical data derived from images in panel A.

To determine if a physiological expression of PD-L1 in the tumor tissue is sufficient to facilitate monitoring with 2c2s, animals were inoculated with PD-L1-positive ccRCC cells under the capsule of the kidney (786-O/Luc cell line with constitutive luciferase expression). Tumor development was monitored by ultrasound. Cy5.5-conjugated ssDNA aptamer probe was systemically administrated by intraperitoneal injection and homing of the marker was followed using whole body imaging (Figure 9A). Cy5.5-2c2s selectively highlighted the ccRCC tumor immediately after systemic administration and did not react with the adjacent tissues. The signal peaked 5 min post-injection and gradually diminished being still clearly distinguished from the background at 50 min post-injection and barely detectable at 80 min post-injection (Figure 9C), demonstrating complete clearance of the probe from the system. Importantly, no signal was observed after administration of Cy5.5-labeled nonspecific ssDNA sequence (Figure 9B3) demonstrating the selective nature of 2c2s accumulation in the tumor. After the aptamer signal was quenched, the animals were administered luciferin to precisely locate the tumor. The strong correlation between tumor location (luminescence) and a signal from a PD-L1-specific aptamer (fluorescence) was demonstrated (Figure 9B1,B2) indicating that indeed the 2c2s aptamer is capable of detecting PD-L1-expressing tumors in vivo.

## 3. Discussion

The spectacular success of PD-L1 targeting monoclonal antibodies in cancer therapy has kindled significant interest in investigating the possible utility of PD-L1 as a diagnostic marker for imaging, a selection marker for antibody therapy [15] and for monitoring of treatment. Antibodies provide suitable tools in some imaging approaches, but aptamer-based detection methods could provide superior in vivo properties and significantly lower production costs.

Aptamer probes are synthetic oligonucleotides that can be easily modified and chemically coupled to reporters suited for various imaging techniques. Positron emission tomography (PET), computed tomography (CT), magnetic resonance imaging (MRI) and fluorescence imaging all require specific probes. Near-infrared imaging (NIR) is especially attractive in this context as it does not require expensive instrumentation and does not expose the patients and the personnel to harmful radiation. Aptamer-based NIR probes could selectively highlight lesions to facilitate diagnosis or surgical resection [16]. Targeting probes might also serve to direct delivery of bioactive agents (photosensitizers, drugs).

In this work, using the SELEX procedure we identified DNA aptamers specifically recognizing the human extracellular domain of PD-L1. During selection, we observed an unexpected profile of affinity maturation. The first five selection steps gradually increased the affinity of the DNA pool, but further steps resulted in decreased affinity. Deep sequencing of aptamer pools at consecutive selection rounds allowed to rationalize the observation. Starting from cycle 6, sequences characterized by weak binding to the target protein prevail. We believe that preferential amplification of aptamers characterized by less stable secondary structures may provide a likely explanation; however, this was not tested systematically. Regardless of the mechanistic explanation, the results allow us to conclude that, paradoxically, limiting the number of selection cycles may be beneficial in selecting high affinity binders. Therefore, fast convergence of selection was sought by manipulating the selection pressure to avoid progressing into cycles favoring highly amplifiable low-affinity binders. The selection of binders from early selection cycles was further facilitated through deep sequencing and sequence cluster analysis.

The selected aptamer, 2c2s, demonstrated an affinity for both the short (single domain) construct used in selection, but also for a two-domain construct of PD-L1 which it did not encounter during selection signifying that we selected a universal PD-L1 detecting binder, rather than an assay-specific molecule. Switching to the cellular environment, the simple system where hPD-L1 is expressed at the surface of CHO cells demonstrated partial masking of the antigen recognized by 2c2s. This could significantly limit the utility of the 2c2s molecule in tumor labeling, was it not for the fact that masking was only characteristic for that particular cell line and possibly related to its hamster origin. All human cancer cell lines tested were characterized by uniform labeling and demonstrated the correlation between α-PD-L1 mAb and 2c2s labeling, indicating that 2c2s is suitable for detection of PD-L1 at the surface of cancer cells.

Given the limited accessibility of the 2c2s epitope on PD-L1 aAPC/CHO-K1 cells, the aptamer was surprisingly efficient in staining the PD-L1 aAPC/CHO-K1 tumor areas in biopsy samples. One could easily distinguish the tumor from the surrounding tissue demonstrating the utility of 2c2s in ex vivo sample analysis.

Earlier reports have already provided aptamers targeting the PD-1/PD-L1 immune checkpoint. Tian Gao et al. [17] obtained an aptamer binding to the PD-1 and antagonizing its interaction with PD-L1. Wei-Yun Lai et al. [18] developed anti-PD-L1 molecules antagonizing the interaction between human PD-L1 and PD-1. In contrast to the prior developments, aptamers delivered in this study do not antagonize the PD-1/PD-L1 interaction. In vitro, anti-PD-L1 aptamers were earlier shown to effectively recognize PD-L1 at the surface of the MDA-MB-231 cell line [19]. Moreover, Jiyuan Li et al. [20] reported an anti-PD-L1 aptamer able to detect the target protein ex vivo in normal human tonsils and non-small cell lung cancer tissue. However, to our knowledge, no earlier study reported in vivo effects.

The success of our ex vivo imaging prompted us to evaluate 2c2s in vivo. PD-L1 overexpression has been documented in both primary [21,22] and metastatic tumors [22,23,24]. In vitro detection is well performed with antibodies, and aptamers provide little advantage. In vivo, however, the small size of aptamers compared to antibodies and versatile labeling possibilities promise wider opportunity for tailoring the response. Upon systemic application, 2c2s aptamer labeled with visible light sensitive fluorescent probe allowed for selective detection of the subcutaneous tumor with very simple instrumentation (optic fiber spectrophotometer). The specificity window of 3-times the signal from tumor adjacent skin, obtained without significant optimization of the probe label or dose, demonstrates the significant potential of our approach in diagnostic imaging.

Detection of malignancy located in an internal organ required adjustment of the detection probe for a red-shifted fluorescence. The label adjustment procedure was successful within a week, demonstrating the flexibility of aptamer design for tailoring the labeling applications. Upon systemic application, a tumor-restricted imaging signal was obtained already at a few minutes after administration and lasted up to 20 min, providing enough time for accurate imaging. The result demonstrated the potential of the approach even in the deeper buried tissue.

Neither intravenous nor intraperitoneal probe application resulted in detectable acute toxicity. Aptamers are generally regarded as safe given their chemical nature identical to the body physiological components. Multiple metabolically neutral labels are available, and it is expected that safe aptamer/label combinations may be achieved *via* optimization. Further testing is necessary to select the best labeling techniques, assess the long-term effects, evaluate metabolism and excretion, and test the utility in clinical settings, but our study has paved this exciting road by providing a universal PD-L1 probe characterized by low molecular weight compared to previously available antibody probes.

## 4. Materials and Methods

### 4.1. Protein Purification

The IgV like domain of human PD-L1 (residues 18–134, C-terminal His-tag) and full extracellular domain of human PD-L1 (residues 18–239) were expressed and purified using previously described protocols [25]. In short, *E.coli* BL21 (DE3) were cultured at 37 °C in LB medium until OD_600_ 1.0, induced with 1 mM IPTG, and further incubated at 37 °C. Bacteria were collected by centrifugation, resuspended in PBS and sonicated. Inclusion bodies were collected by centrifugation, washed twice with 50 mM Tris-HCl pH 8.0, 200 mM NaCl, 0.5% Triton X-100, 10 mM EDTA, 10 mM 2-mercaptoethanol, and once with the same buffer without Triton X-100. Purified inclusion bodies were then solubilized in 50 mM Tris pH 8.0, 6M GuHCl, 200 mM NaCl and 10 mM 2-mercaptoethanol and refolded at 4 °C by dilution into 0.1 M Tris pH 8.0, 1 M L-Arg hydrochloride, 2 mM EDTA, 0.25 mM oxidized glutathione and 0.25 mM reduced glutathione. After refolding, the protein was dialyzed 3 times against 10 mM Tris pH 8.0, 20 mM NaCl, concentrated, and purified by gel filtration on Superdex 75 in 25 mM Na_2_HPO_4_, 25 mM NaH_2_PO_4_ pH 6.4, 100 mM NaCl, 5 mM MgCl_2_ and 10 mM KCl or alternatively in 10 mM Tris pH 8.0 containing 20 mM NaCl. Purified protein was flash frozen in liquid nitrogen and stored for further experiments as 0.2 mg/mL stocks at −80 °C.

### 4.2. In Vitro Selection of Aptamers

Selection of aptamers was performed using a single-stranded DNA library (5’-CATGCTTCCCCAGGGAGATG-N_50_-GAGGAACATGCGTCGCAAAC-3′; 50-nucleotide random sequence), synthesized at 0.2 μM scale and purified by HPLC (IBA, Göttingen, Germany). Aptamers were selected for their specific binding to PD-L1 extracellular domain (residues 18–134, C-terminal His-tag). PD-L1 was immobilized at Dynabeads ™ (Thermo Fisher Scientific, Waltham, MA, USA) in a binding buffer (100 mM Sodium Phosphate, pH 8.0, 600 mM NaCl, 0.02% Tween™-20) and washed with the selection buffer (PBS containing 5 mM MgCl_2_, 10 mM KCl and 0.01% Tween 20, pH 7.4). The ssDNA library or ssDNA pool before each subsequent selection cycle were renatured (5 min at 92 °C, 10 min 4 °C, 15 min RT) and suspended in binding buffer (selection buffer supplemented with 40–120 µg/mL yeast tRNA (Invitrogen, Waltham, MA, USA) and 125 μg/ml BSA (BioShop Canada Inc., Burlington, VT, Canada)). Beads with immobilized target protein were added and incubated for 20 min with shaking at 24 °C. To increase the selection pressure during the selection process, the ssDNA concentration (30 to 0.18 µM) and the immobilized protein bead amount (3 to 0.3 ul) were gradually reduced while increasing the competitor concentration (40 to 120 μg/mL yeast tRNA). After incubation, unbound aptamers were removed by washing with a selection buffer using a magnetic concentrator (Invitrogen, Waltham, MA, USA). Subsequently, the beads with the immobilized protein and associated DNA were suspended in 400 μL of PCR mix containing: 1 μM primers (unmodified ss50_For: 5’-CATGCTTCCCCAGGGAGATG-3′ and 5’-phosphorylated ss50_Rev: 5’-GTTTGCGACGCATGTTCCTC-3’), 5 mM dNTP, 2.5 mM MgCl_2_ and 1.25 U/100 uL polymerase Taq (Thermo Fisher Scientific, Waltham, MA, USA). PCR was carried out for 35 cycles consisting of the following steps: 30 s in 95 °C, 30 s in 53 °C and 30 s in 72 °C. The final elongation was carried out at 72 °C for 5 min. The PCR products were then extracted with a phenol-chloroform-isoamyl alcohol (Sigma-Aldrich, St. Louis, MO, USA) and precipitated overnight with ethanol at −20 °C. The DNA pellet was washed twice with 70% ethanol, dried and dissolved in dH_2_O. The obtained dsDNA was digested with 100U of λ exonuclease (Thermo Fisher Scientific, Waltham, MA, USA) to recover the relevant single strand. Digestion was carried out for 1 h at 37 °C with gentle shaking. Digested products (ssDNA) were again extracted with a phenol-chloroform-isoamyl alcohol mixture, precipitated, and dissolved in 100 μL of dH_2_O.

To eliminate the nonspecific binding, the negative selection was performed by incubating ssDNA pool/library with free Dynabeads prior the aptamer selection cycles. During the selection, an enrichment of the aptamer pool with PD-L1 binding sequences was monitored by ELISA (see below). The aptamer pools after the 5th, 6th and 7th selection cycle were analyzed by Next-Generation Sequencing (NGS; Genomed S. A., Warszawa, Poland).

### 4.3. Sequence Analysis

FASTQ files containing demultiplexed sequences after barcode trimming were further processed. Adapters (flanking sequences) were excluded at both ends and, if needed, sequences were reversed. The obtained aptamer sequences were clustered in groups of identical sequences while only considering sequences at least 10 nucleotides long. The groups were compressed to common sequences (later referred to as compressed_seq) and identified by their cardinality. Compressed_seqs were represented as their nucleotide sequences and 10 most frequent compressed_seqs were clustered with the use of k-means algorithm. The optimal number of clusters was determined employing the silhouette method, and silhouette score was computed for 2 ≤ k ≤ 4. Compressed_seqs in each of the obtained clusters were aligned with ClustalW. The alignments were subsequently processed with dnadist and neighbour programs from PHYLIP package to generate (pseudo-) phylogenetic trees representing the relationships between compressed_seqs (process illustrated in Supplementary Figure S1).

### 4.4. ELISA

Affinity and binding specificity to PD-L1 was assessed by ELISA. A 96-well microtiter plate (Nunc, Rochester, NY, USA) was coated with 100 μL of human PD-L1, BSA, or mouse or human serum and incubated overnight, at 4 °C. Unbound protein was removed by washing with selection buffer. Biotinylated aptamers and nonspecific sequences (in the concentration range of 0–2.5 µM) were added to the wells and incubated for 30 min. Unbound aptamers were removed by extensive washing with selection buffer. Then, 100 μL HRP conjugated streptavidin was added to the wells in 1:200 dilution (R&D Systems, Inc., Minneapolis, MN, USA) in selection buffer. After 20 min of incubation, unbound streptavidin was removed by washing and 100 μL of HRP reagent substrate (R&D Systems, Inc., Minneapolis, MN, USA) was added. The reaction was stopped by the addition of 50 μL of 2N H_2_SO_4_. Absorbance at 450 and 570 nm (correction of optical plate imperfections) was determined using an Infinite 200 PRO multimode reader (Tecan Group Ltd., Männedorf, Switzerland).

### 4.5. Immobilization Test

Biotinylated aptamers (4 μM; 2c2s or negative control) were immobilized on Streptavidin Mag Sepharose (GE Healthcare, Chicago, IL, USA) by incubation in PBS for 20 min in RT. After washing with PBS, the beads were incubated with 0,2% BSA (BioShop Canada Inc., Burlington, VT, Canada) in the SELEX buffer for 30 min at RT to block unspecific sites and washed with SELEX buffer. Next, the beads were suspended in SELEX buffer containing 40 μg/mL tRNA and incubated with PD-L1 (18–239 or 18–134 C_term_ Histag; final concentrations: 90, 45, and 22,2 μg/mL) for 20 min at RT while mixing continuously. After incubation, the beads were washed with SELEX buffer and the bound protein was eluted by boiling briefly in the loading buffer (3% SDS, 10% glycerol, 12,5 mM Tris-HCl, 100 mM DTT, 0,05% bromophenol blue, pH 6.8). The recovered proteins were analyzed by SDS/PAGE. Coomassie blue-stained gels were imaged using ChemiDoc (Bio-Rad Laboratories, Inc. Hercules, CA, USA).

### 4.6. Cell Culture and Binding Assay

To validate the binding of the aptamers to PD-L1 expressed at the cells surface, aAPC/CHO-K1 cells and PD-L1 aAPC/CHO-K1CHO cells stably expressing hPD-L1 protein (Promega), human melanoma cells WM115 and WM266.4 (ATCC CRL-1675 and ATCC CRL-1675), human glioblastoma LN18 (ATCC CRL-2610) and 786-O renal adenocarcinoma (JCRB Cell Bank, JCRB1397) cells were used. Cells were cultured at 37 °C with 5% CO_2_ in an appropriate culture medium supplemented with 10% FBS (InvivoGen) and 1% penicillin/streptomycin. aAPC/CHO-K1 and PD-L1 aAPC/CHO-K1 cells were grown in DMEM-F12, melanoma cells were cultured in RPMI-1640, LN18 in DMEM (4.5 g/L glucose) and 786-O in RPMI-1640. PD-L1 aAPC/CHO-K1 cells medium was additionally supplemented with 50 µg/ml hygromycin B (Sigma-Aldrich, St. Louis, MO, USA) and 250 µg/ml G 418 disulfate salt (InvivoGen, San Diego, CA, USA). Non-transfected CHO cells were used as a negative control.

### 4.7. Flow Cytometry Analysis

To assess aptamer binding to PD-L1 at the cell surface flow cytometry was used. The cells (aAPC/CHO-K1, PD-L1 aAPC/CHO-K1, WM115, WM266.4, LN18 and 786-O) were blocked with HBSS/Dextran solution (Thermo Fisher Scientific, Waltham, MA, USA) containing 10% FBS for 30 min at 37 °C and the FITC-conjugated 2c2s aptamer was incubated with 1 × 10^5^ cells for 30 min in the dark with gentle shaking (180 rpm). Cells were then washed three times with HBSS and harvested with accutase (Thermo Fisher Scientific, Waltham, MA, USA). To confirm the surface expression of PD-L1, cells were incubated with an anti-human PD-L1 antibody (MIH1 clone, Thermo Fisher Scientific, Waltham, MA, USA). Samples were analyzed on a FACSCalibur instrument (Becton Dickinson, Franklin Lakes, NJ, USA; adenocarcinoma cells) using CellQuest software or Guava cytometer (Merck Millipore, Darmstadt, Germany; all other analyses in this study). Data were analyzed in FlowJo v10.8.0.

### 4.8. In Vitro Kinetic Binding Assay

Real-time cell binding assay (RT-CBA) using LigandTracer^®^ Green (Ridgeview Instruments AB, Vänge, Sweden), and a blue (488 nm)-green (535 nm) detector was performed to evaluate the interactions of FAM-labeled 2c2s with PD-L1 protein expressed at the surface of aAPC/CHO-K1 cells. Cells were seeded in each target compartment of MultiDish 2 × 2 (1 × 10^6^ cells). Uncoated free areas of the Petri dish were measured to enable the subtraction of the background signal. The baseline measurement of cells in the absence of labeled aptamer was also performed. Kinetics of binding was analyzed with increasing concentrations (50, 200 or 650 nM) of the labeled aptamer. To evaluate the dissociation process, a fresh cell medium was provided. Affinity calculations (based on the association and dissociation rates) were performed using the TraceDrawer Software (Ridgeview Instruments AB, Uppsala, Sweden).

### 4.9. Fluorescence Imaging In Vivo

The 12-week-old male BALB/c nude mice (mean weight, 16 ± 5 g) were purchased from Anima Lab. Mice were maintained in a pathogen-free environment, on 12/12 night/day cycle, with food and water supplied *ad libitum* throughout the experiments. Weight loss >25% or tumor volume >900 mm^3^ were identified as endpoints for euthanasia. All animal experiments were performed in accordance with institutional guidelines. All experiments were carried out with approval no. 190/2018 of the 2nd Local Institutional Animal Care and Use Committee, Krakow, Poland.

aAPC/CHO-K1 or PD-L1 aAPC/CHO-K1 cells (Promega) suspension in PBS/BD Matrigel Matrix Growth Factor Reduced (1 × 10^7^ cells) was injected subcutaneously into the left flank of each animal. The diagnostics procedures were started when tumors reached more than 0.5 cm in each diameter (which corresponds to tumor volume of about 80–100 mm^3^; up to 21 days after tumor inoculation). For the fluorescence imaging experiment, 2 mg/kg BW 2c2s-Cy5.5 aptamer was administrated intravenously via tail vein injection and then tumors were imaged via fiber optics, and fluorescence spectra from tumor or skin were registered. The measurements were performed up to 96 h post-injection. The fluorescence spectra were collected using a Perkin Elmer LS spectrofluorometer equipped with tumor-imaging-dedicated fiber optics.

### 4.10. Immunohistochemistry and Confocal Imaging

The efficacy of tumor detection with the 2c2s-FAM probe was evaluated ex vivo by immunostaining of tumors obtained from BALB/c nude mice in a prior in vivo imaging experiment. aPD-L1 monoclonal antibody (MIH1 clone, APC conjugated, ThermoFisher Scientific, Waltham, MA, USA) was used as a positive control. aAPC/CHO-K1 and PD-L1 aAPC/CHO-K1 tumors were weighed, snap-frozen and stored at −80  °C. Harvested tumors were cut into 5-μm-thick sections using a cryostat (Leica). Sections were placed on microscope slides for staining. Frozen slides were air dried (30 min) and fixed with ice-cold acetone for 10 min. After acetone evaporation (30 min) slides were washed two times with PBS and then incubated for 10 min in 0.3% H_2_O_2_ in MeOH/PBS. The slides were washed two times (5 min) with PBS and blocked for 1 h in blocking buffer (1× TBS, 0.3% Triton X-100, 5% FBS). The blocking solution was removed and slides were incubated with 2c2s-FAM aptamer or aPD-L1 primary antibody overnight at 4  °C. After incubation, slides were washed three times (5 min) with TBS-0.01% Tween 20, stained with Hoechst33342 for 10 min, rinsed with PBS and mounted with prolong gold mounting medium. Tissues were imaged with fluorescence confocal microscope LSM880 (Carl Zeiss) and the images were analyzed using ZEN software (Carl Zeiss). Five complete and non-overlapping regions of interest (ROI) were randomly selected from each prepared slide. All microscopic images were adjusted with the same parameters.

### 4.11. Orthotopic Kidney Cancer Imaging

Female, 8-week-old Athymic Nude mice were purchased from Janvier Labs, France. The animals were housed and maintained in individually ventilated cages under a 50–60% humidity, 12/12 h light/dark cycle and at 22 ± 2 °C, in SPF conditions at the Faculty of Biochemistry, Biophysics and Biotechnology, Jagiellonian University in Krakow, Poland. All animal experiments were reviewed and approved by the 1st Regional Ethics Committee on Animal Experimentation, Krakow, Poland (approval no: 264/2020).

JCRB1397-786-Luc tumor cells (1 × 10^5^/50µL/kidney) were implanted under the capsule of the kidney using a 1 mL syringe with a 30-gauge needle under anesthesia. Control mice were administrated with PBS into the kidney. Tumor growth monitoring began 4 weeks after cell implantation and continued every 10 days by bioluminescence using an In Vivo Imaging System (IVIS) Lumina (PerkinElmer, Waltham, MA, USA). For this purpose, 0.2 mL (15 mg/ml) D-luciferin (XenoLight D-luciferin potassium salt, PerkinElmer, USA) was injected into the peritoneum of tested mice and visualization was performed after 10 min. The mice were under inhalation anesthesia with 3–4% Isoflurane (Aerrane, Baxter, Poland) throughout the experiment. The 2 mg/kg body weight Cy5.5-2c2s or unspecific sequence (control) labeled with Cyanine 5.5 (Sigma Aldrich, Darmstadt, Germany) was administered systemically by intraperitoneal injection and whole-body imaging using the IVIS 200 Imaging System was performed immediately following and up to 90 min after the injection.

## 5. Conclusions

We delivered an aptameric probe capable of specific detection of PD-L1 expressed on tumor cells both ex vivo and in vivo. We demonstrated that the probe allows for noninvasive tumor imaging, and as such has the potential for further development towards noninvasive diagnostic approaches in humans.

## Data Availability

The data presented in this study are available in the main text and the supplementary materials of this article.

## Supplementary Materials

The following supporting information can be downloaded at: https://www.mdpi.com/article/10.3390/ph15060697/s1, Figure S1: Strategy of sequencing data analysis. Figure S2: Total number of molecules (sequences) in the last three selection cycles in clusters c2, c3 and c4. Figure S3: Effect of 2c2s aptamer on inhibition of PD-L1 and PD-1 protein interaction in vitro. Figure S4: Model of a 2D structure of 2c2s aptamer. Figure S5: The kinetics of aAPC/CHO-K1 and PD-L1 aAPC/CHO-K1 tumor growth. Figure S6: The scheme of fluorescence imaging experiment with PD-L1 aAPC/CHO-K1 tumor bearing BALB/c nude mice.
