# Supplementary material for: Imaging of Clear Cell Renal Carcinoma with Immune Checkpoint Targeting Aptamer-Based Probe
Source: Pharmaceuticals (Basel). 2022 Jun 1;15(6):697. doi: 10.3390/ph15060697 (PMC9229018; doi:10.3390/ph15060697)
Supplement: Supplementary file 1 [file pharmaceuticals-15-00697-s001.zip › pharmaceuticals-1728469-supplementary.pdf]

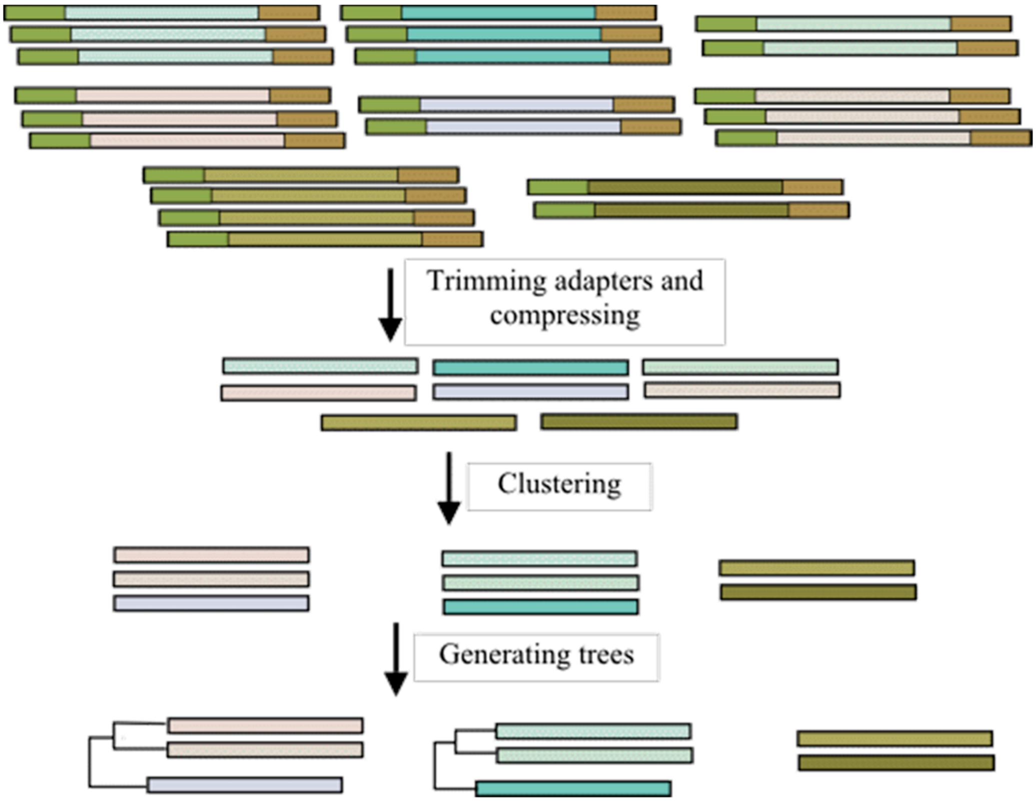

Supplementary Figure S1. Strategy of sequencing data analysis. Raw sequencing data were trimmed of constant regions and identical sequences were compressed into single sequences. These were clustered and (pseudo) phylogenetic trees within each cluster were obtained representing relationships between aptamers.

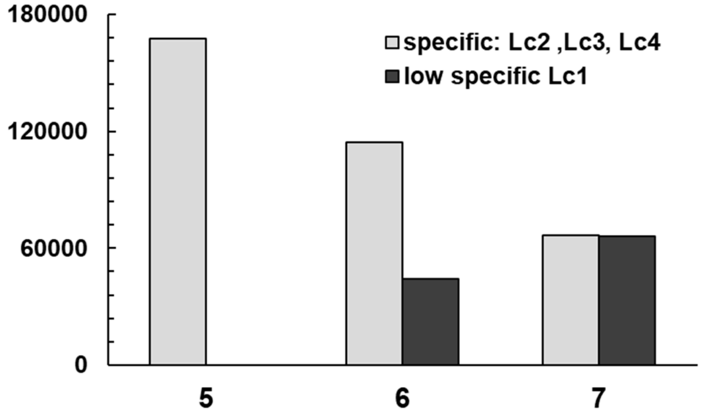

Supplementary Figure S2. Total number of molecules (sequences) in the last three selection cycles in clusters c2, c3 and c4 (showing high binding affinity to PD-L1), and in cluster c1 (with low binding affinity to PD-L1).

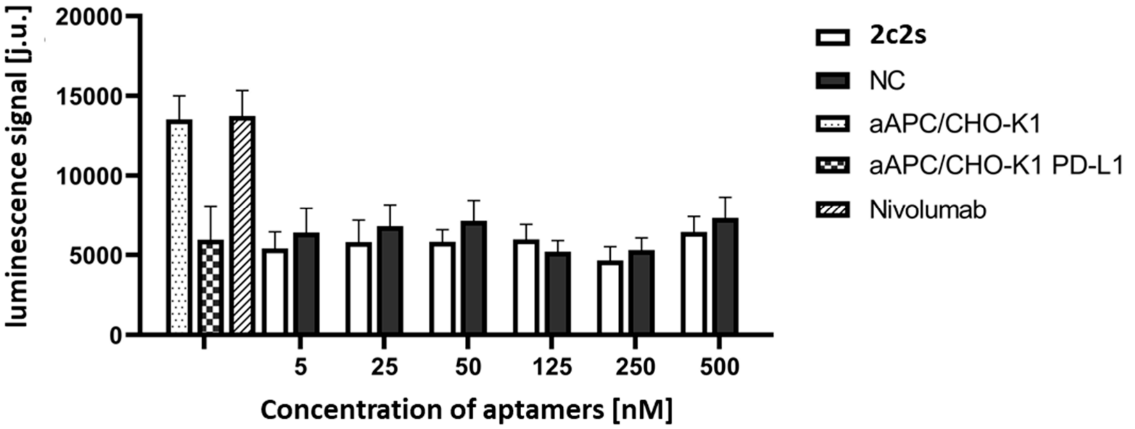

Supplementary Figure S3. 2c2s aptamer does not dissociate PD-L1/PD-1 interaction *in vitro* (reporter assay). The interaction of the PD-1 receptor with PD-L1 leads to the extinction of the luminescence signal. Therapeutic antibody Nivolumab was used as a positive control to confirm dissociation of the PD-L1 / PD-1 complex; aAPC / CHO-K1 - control of luminescence signal in stimulated Jurkat T PD-1 cells; PD-L1 aAPC/CHO-K1- control of the extinction of the luminescence signal in Jurkat T PD-1 cells as a result of the interaction of PD-1 with PD-L1 (basal level); NC- control aptamer with non-specific ssDNA sequence.

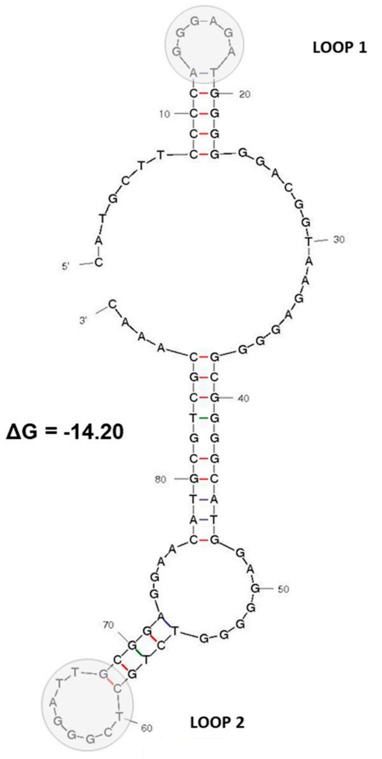

LOOP 1 LOOP 2

CATGCTTCCCCAG**GG**AGAT**GG**GG**GG**AC**GG**TAAGA**GGG**GCG**GGG**CATGGAG**GGG**GTCTGCTC**GGG**ATTGCGGAGGAACATGCGTCGCAAAC

Supplementary figure S4. Model of a 2D structure of 2c2s aptamer. (A) Prediction of 2c2s secondary structure using mFold software http://unafold.rna.albany.edu/results/5/19Sep20-05-54-02/. (B) Sequence fragments responsible for the formation of loops (frames) and a hypothetical sequences creating quadruplexes (bold, underlined).

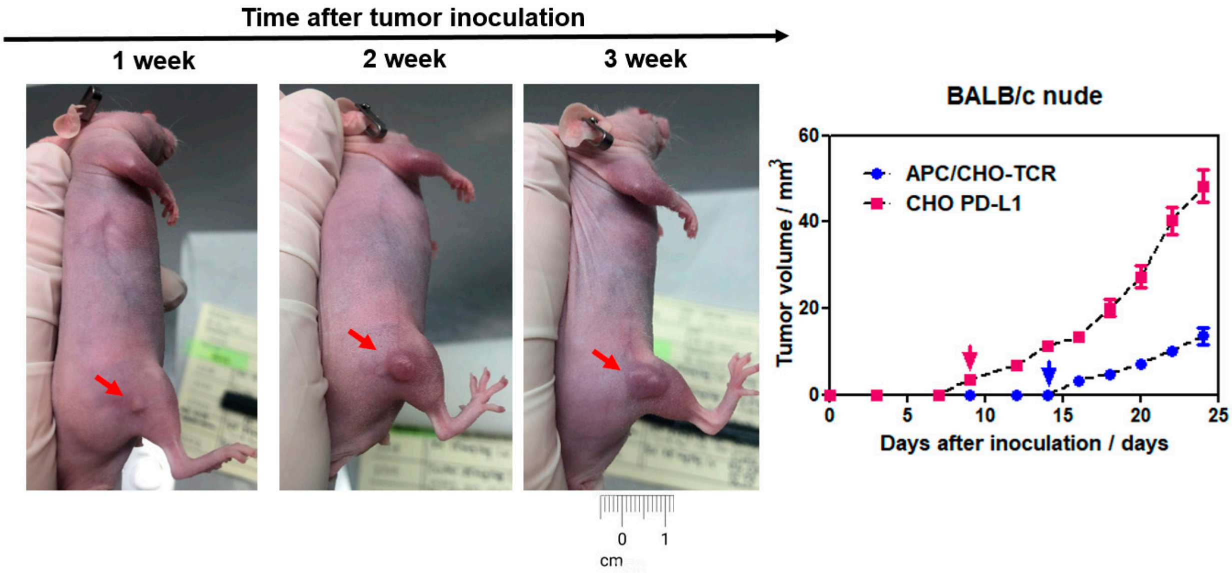

Supplementary figure S5. The kinetics of aAPC/CHO-K1 and PD-L1 aAPC/CHO-K1 tumor growth: BALB/c nude mice bearing CHO-PD-L1 tumors (left) and kinetics data (right).

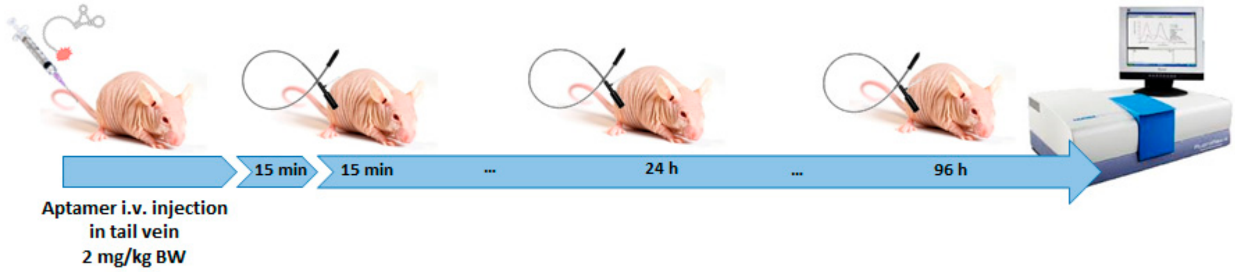

Supplementary figure S6. The scheme of fluorescence imaging experiment with PD-L1 aAPC/CHO-K1 tumor bearing BALB/c nude mice.
